# The Use of Proteins, Lipids, and Carbohydrates in the Management of Wounds

**DOI:** 10.3390/molecules28041580

**Published:** 2023-02-07

**Authors:** Priscilla Barbosa Sales de Albuquerque, Natalie Emanuelle Ribeiro Rodrigues, Priscila Marcelino dos Santos Silva, Weslley Felix de Oliveira, Maria Tereza dos Santos Correia, Luana Cassandra Breitenbach Barroso Coelho

**Affiliations:** 1 Departamento de Medicina, Universidade de Pernambuco, R. Capitão Pedro Rodrigues, 105-São José, Garanhuns 55.295-110, Brazil; 2 Departamento de Bioquímica, Centro de Biociências, Universidade Federal de Pernambuco, Avenida Professor Moraes Rego, 1235 Cidade Universitária, Recife 50.670-901, Brazil

**Keywords:** carbohydrates, healing process, infected wounds, lipids, proteins, wound management

## Abstract

Despite the fact that skin has a stronger potential to regenerate than other tissues, wounds have become a serious healthcare issue. Much effort has been focused on developing efficient therapeutical approaches, especially biological ones. This paper presents a comprehensive review on the wound healing process, the classification of wounds, and the particular characteristics of each phase of the repair process. We also highlight characteristics of the normal process and those involved in impaired wound healing, specifically in the case of infected wounds. The treatments discussed here include proteins, lipids, and carbohydrates. Proteins are important actors mediating interactions between cells and between them and the extracellular matrix, which are essential interactions for the healing process. Different strategies involving biopolymers, blends, nanotools, and immobilizing systems have been studied against infected wounds. Lipids of animal, mineral, and mainly vegetable origin have been used in the development of topical biocompatible formulations, since their healing, antimicrobial, and anti-inflammatory properties are interesting for wound healing. Vegetable oils, polymeric films, lipid nanoparticles, and lipid-based drug delivery systems have been reported as promising approaches in managing skin wounds. Carbohydrate-based formulations as blends, hydrogels, and nanocomposites, have also been reported as promising healing, antimicrobial, and modulatory agents for wound management.

## 1. Introduction to Wound Healing

The skin is a specialized organ that acts as the first line of defense against external damage. It exerts several important homeostatic functions ranging from thermostatic regulation to the repair of flaws in tissue integrity, because of intrinsic or extrinsic factors, through a perfect and coordinated cascade of cells and molecular events, with the aim of rebuilding the injured tissue. The process of tissue repair begins just after the lesion, and is constituted of four distinct stages that overlap in time: hemostasis, inflammation, proliferation, and remodeling [1,2,3,4].

The first stage of wound repair is hemostasis, with the formation of the fibrin clot, stimulation of growth factors, and the reconstitution of the extracellular matrix. The inflammatory phase, characterized by the release of cytokines, edema formation, increased vascular permeability, and the infiltration of neutrophils and monocytes at the wound site, is the second stage. The third stage is characterized by re-epithelialization, with migration of epithelial cells to the edge of the wound, angiogenesis, collagen synthesis, and wound contraction. The final stage is remodeling, characterized by intense matrix production, type 1 collagen remodeling, maturation, and vascular regression; this stage begins 2–3 weeks after injury and can extend for months [2,4].

Simple and complex and acute and chronic are different wound classifications. Acute wound repair is rarely considered as a health problem as the process occurs in a timely and orderly way [5]. During the process, immune-inflammatory cells appear transiently; innate immunity is responsible for removing dead cells and cellular debris, and macrophages and neutrophils participate in the very healing [2]. However, pathophysiological conditions can lead to a deficient repair and the absence of healing after the period of time that would be sufficient for a proper tissue repair [5]. Chronic wounds are usually retained in the inflammatory stage due to the difficulty of progression to the re-epithelialization and dermal remodeling stage [4]. These lesions are characterized by an excess of neutrophils and macrophages [6,7], metalloproteinases [8,9], and Langerhans cells [10] that collectively contribute to healing failure.

Several factors can contribute to the delay of the healing process such as obesity, aging, diabetes, autoimmune diseases, cardiovascular disorders, and burns [11]. The vulnerability of chronic wounds is not completely understood; it is well known that the skin of the elderly and diabetics is more predisposed to deficiencies in the healing process due to reduced hydration, gradual loss of the dermal matrix, increased susceptibility to friction damage, and atrophy [12,13,14]. The high number of people with chronic wounds worldwide generates high costs for public health services since it is estimated that 40 million people have chronic wounds and 1–2% of the population of developed countries will have this type of wound during their lifetime. According to the Markets and Markets report, the global wound care market is expected to reach US$ 22.01 billion by December 2022, which is 24%-fold higher than the amount spent in 2016 (US$ 17.69 billion) [11].

Meticulous tissue handling and wound management with efforts to prevent infection are the most effective alternative to avoid costly wound care since one of the main complications in the wound healing process is its infection. Additionally, the infected wound can be considered a chronic inflammatory state that can increase the mortality rate [15]. Chronic wounds, such as diabetic foot and pressure ulcers, have a greater propensity for the formation of biofilms of pathogenic bacteria since the environment constituted by necrotic tissue and debris allows the attachment of these microorganisms [16]. In view of this, it is mandatory to understand the cellular basis of wound pathology in order to develop therapeutically viable and cost-effective treatments.

Regarding its frequency and the complexity of an impaired wound healing, there is an increased interest in developing natural and innovative approaches for wound repair in order to minimize health costs and enhance treatment efficacy. Different protein-based tools, for example nanostructures, blends, isolated biopolymers, or immobilizing systems, have been studied due to their essential interactions for the healing process [11]. The development of natural lipid-based topical formulations also represents an interesting strategy to manage the wound healing process due to their plasticizing, anti-aging, healing, antimicrobial, anti-inflammatory, and remodeling properties [17].

Carbohydrates are naturally occurring biomolecules that perform a number of key functions in the wound repair process, acting as an energy source, a lubricant, and an immunologic, transport, structural, and regulatory agent [18]. Thus, variations in the composition, structure, and function of some carbohydrates, especially polysaccharides, have been described as developing hydrogels, nanocomposites, and films, among others, for noninfected and infected wound healing management applications.

The purpose of this review is to highlight the skin wound healing, types of wound, and particular characteristics of each phase of the repair process in order to understand the main differences between the normal process and what is involved in the pathological one, more specifically in infected wounds. We also describe recent approaches for wound repair and their importance to enhance the efficacy of treatments by proteins, lipids, and carbohydrates.

## 2. Types of Wound and the Healing Process

Before approaching the healing process, it is essential to define a wound: it is a flaw or an injury in the skin that occurs as a result of different damages, or even as a consequence of physiological conditions, and can extend to other tissues and structures, such as subcutaneous tissue, muscles, tendons, nerves, vessels, and bones [19]. Twenty years ago, Percival [20] reported that there was no standard classification for wounds; however, he predicted a number of different ways in which wounds can be classified, thus helping health professionals in the appropriate injury management. More recently, Tabriz and Douroumis [21] reported their classification into three main categories, including the injury´s cause, the nature of the healing process, and the wound´s clinical appearance. The following paragraphs will detail each type of wound, considering the updated classification, and the multifaceted process involving several inter-related molecular and biological actions directed for tissue regeneration.

In relation to the cause, wounds can occur by mechanical, chemical, radiation, or thermal injuries. Generally, mechanical injuries can be divided into seven types, from superficial to deep wounds, caused by different ways [21]:

(a) wounds by abrasion damage occur in the very superficial part of the skin and heal fast and easily;

(b) punctured/incised wounds are caused by sharp-pointed materials and can evolve to infected wounds;

(c) cut wounds can also be caused by sharp objects, but have blunt additional force;

(d) crush wounds are caused by pressure and blunt force and are usually deeper than abrasions, thus causing more pain and bleeding;

(e) torn wounds are caused by tearing or pulling and can eventually promote an incomplete amputation;

(f) gunshot wounds are closed wounds whose entrance of foreign materials into the body can aggravate and cause the death;

(g) bite wounds can cause torn, ragged, and infected wounds, in addition to bone fractures.

Wounds caused by chemicals or radiation arise from a variety of sources, including electricity, corrosive chemicals, and radiation. The symptoms and severity of the injury depend on the amount (or concentration) of the source, the length of exposure, and the part of the body that was exposed [21,22].

Thermal wounds can be classified into burning or freezing injuries. The first one is divided into four degrees of injury, varying from superficial to deep thickness of the skin. The fourth degree involves the full thickness of the skin in addition to the involvement of other structures such as the subcutaneous tissue and even muscles and bones. Wounds caused by freezing are divided into mild, moderate, or severe type and, contrary to burns, are treated by rewarming the injured part of the skin [21].

Mechanical injuries, for example those caused by external factors such as incision, laceration, abrasion, and ulceration, and also thermal and radiation injuries, are considered acute wounds. With this information, now we can classify wounds by the nature of the healing process; acute and chronic are the two groups of classification. Acute wounds usually heal completely, with minimal scarring according to the normal wound healing process from 8 to 12 weeks after the injury [19,21,23]. Chronic wounds are derived from tissue injuries that heal slowly over more than 12 weeks, and often reoccur [19,21,24].

Regarding more specifically chronic wounds, it is possible to associate them with repeated trauma to the injured area or physiological conditions such as diabetes, persistent infections, and unappropriated treatments. The majority of chronic wounds are assigned to three categories: leg ulcers (caused by venous or arterial deficiencies), pressure ulcers, and diabetic foot ulcers. Chronic wounds are often detained in a self-perpetuating inflammatory stage obtained by a combination of overlapping factors that prevent healing, including impaired angiogenesis/re-epithelialization, dysregulated cytokine/growth factor networks, increased protease activity, and critical bacterial contamination [11,25].

**Figure 1:**
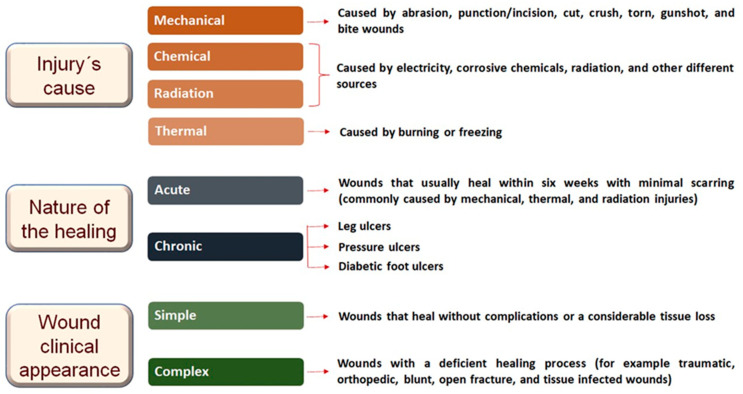
Flow diagram of wound classification.

Considering their clinical appearance, wounds can be divided into simple and complex wounds and this classification provides useful information for effective wound care. Simple wounds heal without complications or considerable tissue loss; they also appear cosmetically aesthetic after healing. Contrarily, complex wounds have a deficient healing process, thus requiring more than conventional treatments, for example simple dressings therapy; in view of this, these well-known difficult wounds, including traumatic, orthopedic, blunt, open fracture, and soft tissue infections, currently have a major socioeconomic impact [21,23]. Correlating the last two above-mentioned wound classifications, both acute and chronic wounds are considered as complex wounds [21,26]. Figure 1 displays a flow diagram of wound classification and summarizes important details of each type of wound.

**Figure 2:**
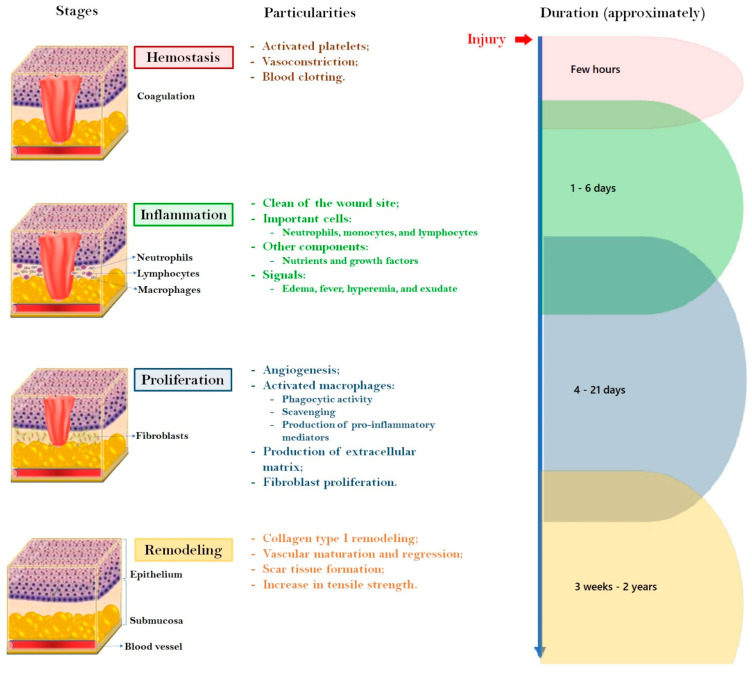
Normal physiological wound-healing process and particular characteristics of its four orchestrated stages (adapted from Albuquerque et al. [27]).

Now we focus on the wound healing, which is a complex biochemical and physiological process consisting of four sequential and well-organized stages: hemostasis, inflammation, proliferation, and remodeling. Platelets, neutrophils, monocytes, and immune, endothelial, and circulating progenitor cells are involved in this sophisticated process with synergic and ordered stages contributing to an efficient wound repair [27]. Figure 2 displays the main particularities for each stage of the normal acute physiological wound repair and the approximate duration of the role process.

The first stage starts within the very first few moments of the injury, where activated platelets contribute to the formation of the fibrin clot through the action of pro-coagulants and the release of prothrombin [28]. This phase is named hemostasis because it is essential to cease the blood flow. In other words, hemostasis is characterized by vasoconstriction of the injured blood vessels and blood clotting, which provides a scaffold for incoming inflammatory cells [29].

The second stage, inflammation, also called inflammatory phase, occurs almost simultaneously with hemostasis, i.e., starts within a few minutes of injury up to 24 h and prolongs to approximately 3 days [21]. Having achieved hemostasis, vascular permeability increases, and inflammatory cells are recruited to the wound site where they opsonize bacteria and remove debris and foreign particles [11]. The first line of defense is composed of neutrophils (or polymorphonuclear cells), which are reported to constitute about 50% of all cells in the wound on the day following injury [30]. They are recruited to attack bacteria and other microbes attempting to invade the body through the open skin wound. Protective mediators, such as antimicrobial peptides, reactive oxygen species, and proteases, are released from neutrophils and directed to the combat, thus generating neutrophil extracellular traps (NETs). Unfortunately, some tissue damage is often observed as a collateral effect from this action [31]. From 48 to 96 h after injury, monocytes are recruited and introduced into the wound site, being named activated macrophages. Lymphocytes are the last recruited cells; they produce antibodies and cytokines/chemokines that mediate survival and the growth of immune cells, fibroblasts, and keratinocytes. Working together, all these components, in addition to growth factors and nutrients, allow the amplification of the inflammation and help the establishment of subsequent healing stages [11,32]. The main inflammation signs, such as edema, fever, and hyperemia are associated with the increased blood flow to the wound site and the accumulation of fluid in the soft tissues. Wound exudate is produced during this stage due to the increased permeability of the capillary membranes. Often, chronic wounds stall in the inflammation phase of healing [21].

As the inflammatory stage ends, the proliferation stage starts with endothelial cells and angiogenesis. One of the key players in the transition from the inflammatory to the proliferation stage is the macrophage (M). They are activated by pathogen-specific molecular patterns and damage-associated molecular patterns, being classified as M1 or M2. In the early stages of wound repair, M1 populations are associated with phagocytic activity, scavenging, and the production of pro-inflammatory mediators. M1 switches to M2 thus allowing the synthesis of more anti-inflammatory mediators and the production of extracellular matrix (ECM) through interactions with the wound microenvironment and mechanical signaling [7,33,34]. The proliferation stage includes an increase in the transport of oxygen and nutrients due to the angiogenesis as well as re-epithelialization, which consists of keratinocytes migration into the wound, followed by its proliferation, and the reconstitution of the dermis by re-structuring the connection between epidermis and dermis. By the fifth day, the maximum formation of blood vessels and the formation of wound contraction is gained by the proliferation of fibroblasts and the differentiation of myofibroblasts into fibroblasts; all these components are characteristics from the granulation tissue. The ability to extend and retract of the new population of fibroblasts, in addition to the attachment of fibroblasts to collagen, shift the wound microenvironment from the inflammatory to the growth state and lead to the foundation of a scar tissue [11,29].

The fourth and final stage is the remodeling phase, whose transformation of collagen is driven from type III to type I. This is also called the maturation phase because the provisional wound matrix is replaced with proteoglycan and collagen, thus reaching an organized and rigid scar tissue [21]. The remodeling stage is quite important for clinical outcomes after the complete closure of the wound. A tightly controlled balance between metalloproteinases and their inhibitors results in the development of a normal scar where cross-linked collagen fibers are rearranged in parallel packages forming strength tension lines. If collagen deposition is too low, the strength of the remodeled skin will be significantly reduced; on the contrary, if collagen deposition is too high, the resulting scar can become hypertrophic or overgrown such as a keloid [11,34].

All these stages culminate with the reorganization of the injury, i.e., the transition from reparative to functional tissue. The tissue tensile strength is restored by the alignment along the tension lines in addition to the degradation of the cells previously reported, especially in the second and third stages of wound healing. In view of the sophisticated repair process, the reactions are synergic and ordered, contributing to a wound repair without interruption, for example, how it occurs in simple and acute wounds [2,35]. Differently, chronic wounds are interrupted by an orderly process, for example, the infection of microorganisms. Now we focus on infected wounds, the main pathogens associated with the infection, and different and recent treatments for infected wounds.

## 3. Infected Wounds

Skin microbiota comprises four prevailing bacterial phyla (Proteobacteria, Actinobacteria, Firmicutes, and Bacteroidetes) capable of forming biofilms and inhibiting skin infections [36]. Furthermore, microorganisms can be found in open wounds, however their presence does not mandatorily represent a wound infection since such infection is characterized by inducing an immune response from the host, generating, for example, erythema, pain, swelling and local inflammation, unpleasant odor, increased exudate, and delayed healing [37,38].

However, when there is an injury to the skin, microorganisms from its normal microbiota as well as some exogenous can infect the wound since the tissue is warm, humid, and rich in nutrients [36]. In wounds that are in the early stages of healing, *Staphylococcus aureus* and methicillin-resistant *Staphylococcus aureus* (MRSA) are the most frequently isolated, while *Pseudomonas aeruginosa* and *Escherichia coli* are commonly detected in chronic wounds since they can infect deeper skin layers. Chronic wound infections are usually polymicrobial; determining the accuracy of the species present in the wound is challenging since it depends on the identification method (standard culturing or molecular methods) and sampling, for example, whether it was obtained by surface swabs, biopsies, or a collection of wound fluid and exudate [39].

A wound infection begins with the contamination and colonization of the local site progressing to biofilm formation and may eventually cause a systemic infection. Biofilm is the community of microorganisms, such as prokaryotic cells, which aggregate to form a colony that are embedded in a layer called an extracellular polymeric substance (EPS) made of polysaccharides, DNA, lipid, and proteins among other molecules, which adhere to each other on an abiotic or biotic surface, such as a wound [40].

**Figure 3:**
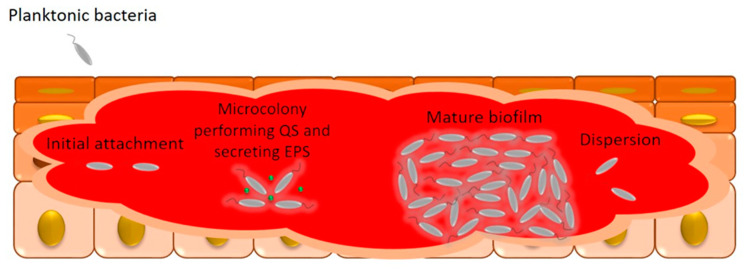
Biofilm formation in wound. Planktonic microbial cells adhere to the wound and form microcolonies in which bacteria begin to secrete molecules to communicate with each other, that is, they perform quorum sensing (QS) as well as secrete extracellular polymeric substance (EPS) in which microbial cells are imbibed until they develop in mature biofilms where microorganisms from the biofilm can detach to infect new sites.

Biofilm formation involves the following steps, which are shown in the Figure 3. (i) Attachment of bacteria to a surface in which the free-floating planktonic cells become sessile; this stage involves nonspecific chemical interactions, such as van der Waals, hydrophobic, and/or electrostatic forces, in addition to adhesins that may be secreted by bacteria or are present in extracellular appendages (pili, flagella, and fimbriae). (ii) Microcolony formation in which bacteria secrete EPS, thus further strengthening their bond with each other and with the substrate, in addition to forming a physical barrier with the extracellular environment. Microorganisms also begin to secrete molecules named autoinducers responsible for cell–cell communication, that is, quorum sensing. (iii) Biofilm maturation where microcolonies progressively increase, recruiting more microbial cells, together with the deposition of organic and inorganic solutes. (iv) Detachment of cells that occurs when some cells detach from the biofilm individually or in clusters to colonize new niches [16,41].

About 60% of chronic wounds require debridement, which has been associated with biofilms, whereas only 6% of acute wounds are associated with the presence of biofilm; the micro-environment of chronic wounds, which contain necrotic debris and a low oxygen tension and are dampened, facilitates microbial growth [42]. Microbial biofilms in wounds can generate a state of chronic inflammation capable of inhibiting re-epithelialization, protecting microorganisms against the host immune response and antibacterial therapies [43].

## 4. Treatments for Topical Infected Wounds

Regarding the most recent treatments for wound healing, many strategies have been developed to reach skin lesion closure, for example, antibacterial ointments, synthetic growth factors, traditional dressings (gauze, films, and foams), polyurethane, three-dimensional bioprinting (stereolithography, microextrusion, laser-assisted, or inkjet printing), and hydrogel bioadhesives (natural, structurally engineered, or synthetic ones) [27,28]. Among them, different types of treatments from different sources (proteins, lipids, or carbohydrates) can be directed for chronic wounds, for example the infected ones.

Considering the limitations associated with conventional in vitro studies, and the reluctance to test, for example, products obtained from nanotechnology on injured patients, animal models are critically important for laboratory investigations into wound healing. Based on in vivo results, it is possible to better understand wound-associated processes and mechanisms [44]. Now, different and recent treatments for wounds are reported by highlighting the source of the treatment (proteins, lipids, or carbohydrates, in macro-, or micro-, and nanoscale), the type of test (in vitro, in vivo, or both), the animal model (if any), the cause of infection (if any), and the results associated with substantial advancements in injury therapies.

### 4.1. Proteins

Tissue repair, also named fibrogenesis, is a complex process orchestrated by different cell lines (inflammatory cells, epithelial cells, and myofibroblasts) and the extracellular matrix, in response to the wound healing process [45]. Various proteins are important actors mediating cell–matrix and cell–cell interactions. For example, integrins facilitate the communication between non-parenchymal cells of the extracellular matrix, such as inflammatory cells and fibroblasts, and parenchymal cells [46]. Recent research results show that defensins are known for their excellent antibacterial activity, thus playing an essential role in the complex pathophysiological changes of diabetic wounds [47]. Collagen, one of the most abundant proteins in the extracellular matrix, is an important source of elasticity and strength in the extracellular matrix and contributes to the structural and physiological integrity of tissues. This protein plays an important role in regulating the wound healing process by attracting fibroblasts and supporting the formation of new tissue in the wound bed [48].

Silk sericin (SS), one of the two major proteins forming the silkworm cocoon, in this case the *Bombyx mori* cocoon, was used to synthesize a hydrogel by repetitive freezing–thawing. SS, poly (vinyl alcohol) (PVA), and azithromycin (AZM) were crosslinked with genipin (GNP) and tested towards *S. aureus*, *P. aeruginosa*, *E. coli*, and *Candida albicans*. The hydrogel also showed sustained SS and AZM releases, cytocompatibility on keratinocytes and fibroblasts, as well as skin adhesion ability when freeze-dried. Regarding specifically the in vivo study using an infected mouse full-thickness burn model with a 10% total body surface area, the hydrogel presented a better burn wound healing effect than the commercial Tegaderm™ film dressing, thus minimizing systemic burn effects [49].

Antibacterial hydrogels, such as the above-mentioned AZM-hydrogel composed of SS, PVA, and GNP, are receiving increasing attention in the aspect of bacteria-infected-wound healing. However, biofilm infections and bacterial drug resistance leads to the hard healing of wounds, thus the elaboration of alternatives that can circumvent these issues is essential. For example, Ouyang et al. [50] cross-linked the green industrial microbicide tetrakis (hydroxymethyl) phosphonium sulfate (THPS) with model protein bovine serum albumin (BSA) to form an antibiotic-free protein hydrogel with excellent biocompatibility and superior antibacterial activity against drug-resistant bacteria and biofilms. When tested as a wound dressing in an in vivo study, the BSA-Hydrogel accelerated the reepithelization of MRSA-infected skin wounds without inducing significant side effects. The results were reported as a way to provide a facile, feasible, and general gelation strategy with promising applications in hospital and community MRSA disinfection and treatment.

Another silk hydrogel was developed with the purpose to avoid polymicrobial infections in infected diabetic wound ulcers. The dressing was produced with the silk hydrogel and a combined therapeutic effect of metal chelating dipeptide (L-carnosine) and curcumin. The results from cell viability revealed that the designed hydrogel matrix was compatible to human cells and significantly accelerated the diabetic healing potential. Additionally, the hydrogel presented bacterial inhibition against *S. aureus* and *E. coli* via in vivo mice wound sites, which indorsed the diabetic wound healing efficiency in streptozotocin-tempted diabetic mice [51].

Another example of nanocomposite hydrogel was fabricated with oxidized alginic acid, dopamine, and antimicrobial peptide ε-polylysine. The system was then cross-linked with acrylamide and tested against gram-positive and gram-negative bacteria; its hemostatic and adhesive properties were also tested. The designed hydrogel showed excellent bacterial inhibition and, when compared with a commercial alginate sponge, accelerated the healing of infected full-thickness wounds by reducing inflammation and promoting angiogenesis and collagen deposition. Therefore, the authors highlighted the nanocomposite as a multifunctional dressing for promoting the healing of infected wounds [52].

Gelatin is the hydrolysis product of the well-known collagen protein derived from connective tissues in animal skin, bone, and tendon. Due to their biocompatibility, convenience for chemical modifications, and degradability, gelatin-based hydrogels have a broad range of biomedical fields. However, their poor antibacterial ability hinders the application of gelatin hydrogels in treating infected wounds. Tao et al. [53] developed a series of multifunctional hydrogels based on gelatin methacrylate (GelMA) and dopamine methacrylate (DMA), both of them immersed into zinc nitrate solutions. The obtained wound dressings showed strong antibacterial activity against *E. coli*, good cytocompatibility, and enhanced adhesion, proliferation, and migration of NIH-3T3 cells. With the promising results, authors described Zn-incorporated hydrogels as bioactive materials potentially suggested as wound dressings for infected full-thickness wound healing and skin regeneration.

A gelatin hydrogel incorporated with bio-nanosilver (silver nanoparticles from a spent mushroom substrate) functionalized with lactoferrin (LTF) was prepared as a dual-antimicrobial action dressing. It was synthesized, characterized, and tested against *S. aureus* and *P. aeruginosa*, thus demonstrating an adequate release of nanoparticles and LTF, with promising antimicrobial effects against both bacterial strains. The authors concluded that the system was successfully synthesized as a new approach for fighting biofilms in infected wounds and thus may be applied to accelerate the healing of chronic wounds [54].

Not only collagen-containing systems but collagen itself is being prepared to boost its efficacy in wound healing. With presentations varying from particulate to powdered forms, this protein has been also tested as an adjunctive therapy for chronic wounds with indications, limitations, and principles of use. In general, the scientific literature reports a need for high quality studies and randomized control trials to support its use in clinical practice [55].

Different strategies involving biopolymers, nanotools, blends, and anti-inflammatory and antimicrobial immobilized drugs have been studied in nanofibers, nanoparticles, hydrogels, films, and sponges based on collagen. They have been recently reported as efficient alternatives in the healing of wounds, infected or not [48]. For example, a bilayer membrane composed of collagen, chitosan, *Aloe vera*, and gelatin was tested on a full-thickness skin wound model. The results of the evaluation revealed that the fabricated asymmetric membrane could facilitate wound healing by enhancing cellular activities and collagen deposition in addition to promoting proliferation within 10 days of treatment [56].

Fibrin, an important plasma protein involved in the coagulation process, was used as a hydrogel basis for the incorporation BNN6-loaded mesoporous polydopamine nanoparticles. After administration, the hydrogel created local hyperthermia and released large quantities of NO gas to treat the MRSA infection. Furthermore, the fibrin and small amount of NO originated from the hydrogel action promoted wound healing in vivo [57].

The use of peptides is an emerging alternative to conventional treatment strategies, including oral or parenteral antibiotics, in the treatment of wound infections. Antimicrobial peptides (AMPs), for example, are natural antimicrobials produced by plants, animals, fungi, protozoa, and bacteria that own potent antibacterial and wound healing effects. They are able to develop a sustained delivery approach for the control of the bacterial growth at the wound site; however, some limitations are reported (for example, instability related to oxidation, hydrolysis, and proteolysis) and then associated with a low residence time for topical applications to the wounds [58,59]. One way to circumvent these issues is the incorporation of AMPs in delivery platforms such as hydrogels [60]; their three-dimensional network, usually based on hydrophilic polymers, can enhance peptide stability and antimicrobial activity, as was reported by Thapa et al. [59] working with a hybrid hydrogel composed of Pluronic F127 (PF127), ethylenediaminetetraacetic acid (EDTA) loaded liposomes, glutathione (GSH), and the AMP from *Lactococcus garvieae*. Potent in vitro antibacterial and anti-biofilm effects were demonstrated against *S. aureus*, while the in vivo treatment of MRSA infected mouse wounds suggested potent antibacterial effects.

Nisin, the first known peptide with antimicrobial activity isolated from *L. lactis* in 1947, was purified, encapsulated in lipid nanoparticles, and then incorporated in a composite hydrogel wound dressing based on natural polysaccharides [gellan gum (GG) and a mixture of GG and alginate]. The most effective antimicrobial activity against *S. pyogenes* was observed for GG and nisin-loaded lipid nanoparticles [61]. AMPs derived from collagen were already obtained from marine sources, such as collagencin, which is derived from fish collagen [62].

An AMP-modified hyaluronic acid (HA-AMP) was prepared in a hydrogel system through Schiff’s base formation between the aldehyde group of oxidized-dextran and the amino group HA-AMP and platelet-rich plasma, and tested for the treatment of chronic infected wounds. The system exhibited significant inhibition zones against three pathogenic bacterial strains (*E. coli*, *S. aureus*, and *P. aeruginosa*) and the slow release of the modified AMP. The in vivo evaluation demonstrated that the hydrogel could significantly improve wound healing in a diabetic mouse infection by regulating inflammation and accelerating collagen deposition and angiogenesis [63].

Other differently sourced proteins were reported by the scientific literature dealing with wound healing therapy. For example, albumin, the most abundant plasma protein, essential in maintaining the osmotic pressure of plasma and transporting endogenous compounds (pigments, other proteins, and cholesterol), had its release behavior from the nanoparticles studied, and the suggestion for the on-demand antibacterial release upon application to infected wounds. Zein, a plant protein found in maize endosperm composed of four nonpolar amino acids (glutamine, leucine, proline, and alanine), was suggested as potent topical antibacterial formulations. However, no commercial products of zein for the topical antibacterial therapy of infected wounds were reported [64]. Therefore, considering the above-mentioned recent publications, proteins could be considered promising strategies for the development of formulations to treat wounds, including the infected ones.

### 4.2. Lipids

Lipids perform essential biological functions within the body, as a source of energy, as structural components of cell membrane, and as signaling molecules; their composition is associated with dietary fatty acid consumption. In the context of wound healing, lipids provide energy to the wound repair; they are responsible for providing substrates from lipids for wound cell proliferation and remodeling, highlighting that the consumption of adequate lipids is essential to the wound healing process [17]. In addition, lipids act as signaling agents during wound healing and tissue regeneration.

Many studies have reported the use of lipids, especially fatty acids, to manage wound healing [65,66]. Fatty acids are carboxylic acids with a long hydrocarbonate chain that compound the structural components of oils and fats, among other categories of lipids [67]. Vegetable oils are known to be a low-cost source of unsaturated fatty acids for the production of topically administered pharmaceutical products. Studies have reported the evaluation of omega-3, omega-6, and omega-9 in their free form and as blends on immune cell functions [68], and edible vegetable or animal oils on wound healing processes [69,70].

Vegetable oils have been reported to promote beneficial effects in cutaneous skin and especially in the wound healing process since their antimicrobial, antioxidative, and anti-inflammatory activities stimulate cell proliferation, reepithelization, and the reconstruction of the skin’s lipid barrier [70,71]. For example, virgin coconut oil was shown to be potent and fast healing when topically applied to treat excision wounds in young rats, evidenced by a decreased time of complete epithelization; significantly increased levels of total collagen, elastin, sialic acid, hexose, and DNA; a low level of lipid peroxidation; and an increase in fibroblast proliferation in treated wounds compared with control wounds [71]. Previous studies reported that coconut oil is rich in fatty acids of a medium chain length (6–12 C) [72], and these molecules may act in modulating cellular proliferation and cell signaling [73]. Thus, the wound healing properties of virgin coconut oil may be attributed, in part, to its fatty acids’ components. Other studies evaluated surgically induced wound closure evolution among rats treated with two oil blends: sunflower/canola oils 85/15 (BL1) and canola/linseed oils 70/30 (BL2); BL1 being administered during the inflammation phase (days 0–3), and BL2 in the tissue formation and remodeling phase (days 4–15). When compared with the control group treated with physiological saline, an increase in the areas of wounds treated with the blends in the inflammatory phase was observed, probably associated with the pro-inflammatory properties of the n-6 fatty acids present in BL1. Secondly, a steeper closure curve was observed, signaling a faster healing wound process resulting from the initial exacerbation of the inflammatory stage with the anti-inflammatory effects of the n-3 fatty acids from BL2 [74].

Beneficial effects for skin wound healing were also observed for *Sapindus mukorossi* seed oil, abundant in monounsaturated fatty acids. Wounds were created on the dorsum of rats and treated with a hydrogel based on carboxymethyl cellulose (CMC)/hyaluronic acid (HA)/sodium alginate (SA) for releasing the *S. mukorossi* seed oil [75]. Treated wounds exhibited an acceleration of the sequential skin wound healing process, including a higher decrease in size when compared with the untreated control. In addition, the oil showed significant anti-microbial activity against *Propionibacterium acnes*, *S. aureus*, and *Candida albicans*. Ferreira et al. [76] developed a biocompatible gel of chitosan associated with buriti oil (*Mauritia flexuosa* L.) to evaluate its potential to heal skin wounds. The lipid composition of buriti oil was also investigated, and it is basically composed of oleic and palmitic acids. Although buriti oil did not show antimicrobial activity, the composite chitosan-buriti oil evidenced potent action against *S. aureus* and *Klebsiella pneumoniae* at the 10 mg/mL. In addition, the composite gel also showed antioxidant activity and promoted faster and complete wound retraction, suggesting its potential for treating infected skin wounds. An oil extracted from *Opuntia ficus indica L. inermis* (OFI), composed by unsaturated fatty acids, triacylglycerols, phytosterols, and tocopherols [77], also demonstrated antimicrobial and wound healing potential [78]. An in vitro significant antimicrobial effect was observed against *Enterobacter cloacae*, *C. parapsilosis*, *C. shake*, and against the opportunistic *Penicillium*, *Aspergillus*, and *Fusarium*. OFI oil also showed potent wound healing, preventing cutaneous infections, and reducing the re-epithelialization phase in a rat model, suggesting a potential approach to treating infected wounds.

In the research to improve wound healing mechanisms, biological membranes have been evaluated to accelerate the healing, preventing infection and sepsis [79]. Frog (*Rana tigerina*) skins have shown a significant role in wound healing to induce proliferative activity of the epidermal and dermal cells. Frog skin lipid content is predominantly phospholipids and exhibits a dose dependent acceleration of wound healing, more specifically on acute inflammation and immunostimulatory response, whether topically applied or injected intraperitoneally [80].

**Figure 4:**
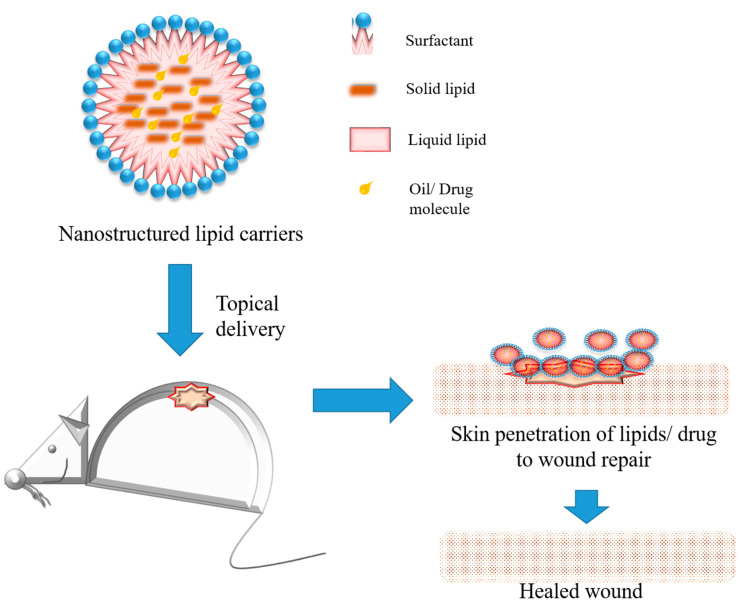
Schematic representation of nanostructured lipid carriers, their composition, and application for wound healing on rat model.

Considering topical skin preparations that are commercially available, frequently they show the same limitations to impair wound healing, such as toxicity, drug instability, and a limited time of action [81]. Recently, topical forms containing nanostructured lipid carriers (Figure 4) represent a strategy to develop new products for wound care, mainly drug delivery systems, dressings, and films [17]. Considering the healing, antimicrobial, and anti-inflammatory properties of lipids, added to their solubility, stability, and plasticizing action, the lipids into formulations increase skin penetration and release the profile of the drugs, improving wound healing therapies compared with conventional treatments [66]. One research has reported the development of nanostructured lipid carriers based on cocoa butter as a solid lipid and olive oil as a liquid lipid to be loaded with eucalyptus oils and to be used to improve wound healing [82]. The surfactant chosen to stabilize the nanostructures was lecithin. Wound healing properties towards normal human dermal fibroblasts in vitro, and wound healing in vivo on a rat burn model, were characterized. These nanostructured lipid carriers showed good bioadhesion and cytocompatibility, adding to the enhancement of the in vitro and in vivo wound healing process and antimicrobial activity against *S. aureus* and *S. pyogenes*, two gram-positive bacteria commonly present in the skin. In this context, it is suggested that the high content of oleic acid, already known for its proliferation enhancement potential, associated with eucalyptus oil, promoted a synergic effect to wound repair and antimicrobial activity [83].

Other studies evaluated the effect of lipid nanocarriers containing omega-3 fatty acid or liposomes containing omega-3 fatty acid and resveratrol in cotton textile substrates as dressings for wound healing. Considering the role on inflammatory response, both formulations inhibited nitric oxide (NO) production, demonstrating an anti-inflammatory effect that improved the wound healing process [65]. Films containing lipids have been developed for application in wound care, for example, chitosan films containing oleic acid or linoleic acid and glycerol [84], that were tested on wounds in burned patients, and it was evidenced that glycerol contributed to film adhesion, and the film promoted good epithelialization in a period of 12 to 15 days. Another chitosan film was prepared containing thyme oil for use as a healing dressing that showed potential antimicrobial and antioxidant activities, and was a promising approach for wound healing dressings [85].

In bacterial infected wounds there often occurs a disruption in the wound healing process, which is a challenge to treat, mainly on chronic pressure ulcers and diabetic wounds. Researchers have demonstrated that the antimicrobial lipids are broad-spectrum antibacterial agents, acting as immune innate responses and by specific mechanisms. Antimicrobial fatty acids act in the bacterial membrane, and the long-chain bases may inhibit cell wall synthesis [86]. Kim et al. [87] described the wound healing properties of a mix of 10 major lipid components of the *Chamaecyparis obtusa* extract. The 10-lipid mixture showed bactericidal effects against *S. aureus* and *S. pyogenes*, and protective effects against staphylococcal α-toxin-induced keratinocyte cell death. In addition, the 10-lipid mixture accelerated the healing of wounds superinfected with *S. aureus* in vivo.

Ghodrati, Farahpour, and Hamishehkar [88] conducted a study to evaluate the efficiency of peppermint essential oil (PEO) loaded into nanostructured lipid carriers (PEO-NLC) on in vitro antibacterial activity and in vivo infected wound healing in mice models. PEO and PEO-NLC demonstrated antibacterial activities against *S. epidermidis*, *S. aureus*, *Lysteria monocytogenes*, *E. coli*, and *P. aeruginosa* in vitro. In vivo analysis showed a greater wound contraction rate, fibroblast infiltration, collagen deposition, and re-epithelialization in PEO and PEO-NLC-treated animals when compared with the control group. Thakur et al. [89] developed nanoengineered lipid-polymer hybrid nanoparticles with chitosan encapsulating the steroidal antibiotic fusidic acid to evaluate its drug release and permeation, antibacterial activity, cytotoxicity, and skin safety profile for the management of wound infections. The developed nanocarriers offered sustained drug release and enhanced drug permeation; a safe profile on HaCat cell lines, suggesting non toxicity; and a reduction of five-times and four-times its inhibitory concentration was observed against MRSA 33,591 and methicillin-susceptible *S. aureus* (MSSA) 25921.

Microalgae *Spirulina platensis* is recognized as a source of lipid and polyunsaturated fatty acids, and free fatty acids were isolated to explore their antimicrobial properties for skin diseases, focusing on *Candida* species biofilm related to chronic wounds [90]. The lipid extracts were vectorized using a macroalgal-alginate nanocarrier and exhibited a good anti-biofilm activity (about 50% inhibition after 24 h at 0.1 mg/mL), and the safe application of keratinocytes, suggesting compatibility with a topical use. When enriched with spirulina, the anti-biofilm activity of lipid extracts was potentiated at low concentrations (about 80% inhibition after 24 h at 0.2 mg/mL), representing a new approach against microbial biofilm formation on chronic wounds. Thus, lipids represent a potent strategy for producing topical formulations to treat infected wounds.

### 4.3. Carbohydrates

The wide variety in the composition, structure, and function of naturally occurring carbohydrates and their derivatives amplifies the physical, chemical, and biological properties interesting in wound treatments, such as biocompatibility, bioadhesiveness, antibacterial potential, injectability, and drug delivery [91]. For example, chitosan is a non-toxic, antioxidant, and antimicrobial polysaccharide that has been reported to promote healing effects on infected wounds [92]. A study evaluated the effects of the topical co-administration of chitosan and platelet-rich plasma (PRP) on the infected burn wounds model by *C. albicans* in Wistar rats. Experimental groups were organized to receive any agent (control), clotrimazole (clotrimazole group), PRP (PRP group), and chitosan + PRP (chitosan + PRP group), and the wound healing processes were evaluated. The chitosan + PRP group showed a higher decrease in the wound size when compared with other groups, and antioxidant activity was improved in all the treated groups compared with the control group. It suggests a promising role of the topical application of chitosan and PRP in the infected burn wounds [93].

Another example is an exopolysaccharide (EPS-S3) isolated from a marine bacterium *Pantoea* sp. YU16-S3, investigated for its wound healing properties [94]. EPS-S3 showed biocompatibility with dermal fibroblasts and keratinocytes, and promoted cell adhesion, cell proliferation, and cell migration in vitro, while in vivo the exopolysaccharide induced the re-epithelializarion of injured tissue in rats, demonstrating its potential for cutaneous wound healing applications. The potential use of exopolysaccharide (EPS) produced by *Nostoc* sp. strains PCC7936 and PCC7413 in wound healing was evaluated by Alvarez et al. [95]. Both EPS may form biocompatible hydrogels and promote fibroblast migration and proliferation, and are attractive for the application in skin formulations to treat injuries.

**Figure 5:**
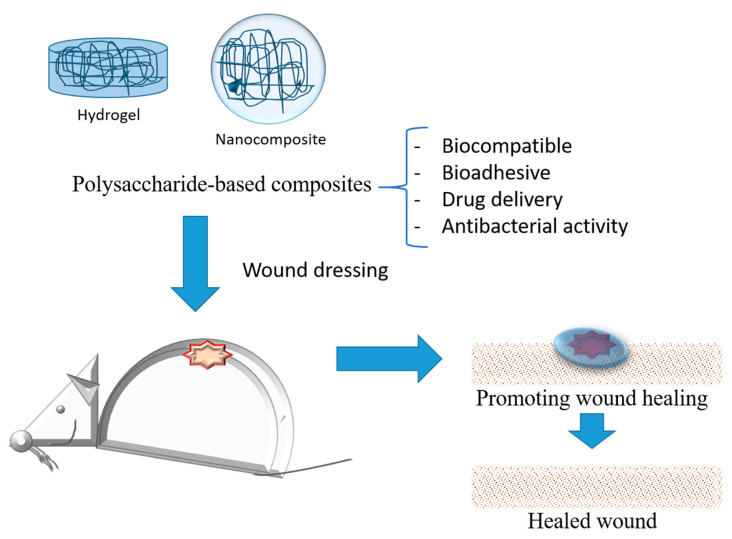
Schematic representation of polysaccharides-based composites applied as wound dressing for treatment.

Typical modifications such as polymerization, etherification, oxidation, crosslinking, and graft copolymerization have been explored to develop modified carbohydrates, especially polysaccharides, and have been potential candidates for wound care. Polysaccharides-based hydrogels and nanocomposites (Figure 5) show a three-dimensional network and other beneficial properties of interest for the delivery of different formulations for oral and topical applications [96].

However, a huge challenge worldwide is to treat infected wounds, mainly non-healing chronic wounds, encouraging the use of natural polymers such as carbohydrates to create new formulations for the treatment of skin infections. In the pharmaceutical area, polysaccharide-based biomaterials have promoted controlled release and adhesion, mechanical protection, cytocompatibility, and antibacterial action, all properties of interest for infected wound healing [97]. Archana et al. [98] developed a nanocomposite based on chitosan, poly(N-vinylpyrrolidone) (PVP), and titanium dioxide (TiO_2_) nanoparticles that demonstrated excellent antimicrobial efficacy against pathogenic bacteria and good biocompatibility, adding to an acceleration of the healing process of open excision type wounds in albino rat models, with reductions in the open wound area from day 3. Another study reported the development of porous sponge dressings based on water-soluble thymine-modified chitosan (TC) derivatives with degrees of substitution ranging from 0.23 to 0.62 [99]. TC derivatives showed broad-spectrum antibacterial activities against gram-negative bacteria, gram-positive bacteria, fungi, drug-resistance bacteria, *P. aeruginosa*, and *Acinetobacter baumannii*. TC sponge dressings promoted the faster regeneration of epithelial tissue, collagen deposition, and new blood vessel formation speed, promoting the wound healing process.

Laser burn wound healing and the anti-inflammatory activity of a polysaccharide preparation from *Pimpinella anisum* seeds (PAP) were investigated using a carrageenan-induced paw edema model in mice [100]. PAP exhibited significant antioxidant, anti-inflammatory, and antibacterial activities, evidenced by a reduction on edema, cellular infiltration, and oxidative stress markers. A gel based on PAP was also developed and topically applied on laser burn lesions, and induced wound contraction, re-epithelization, and remodeling phases after seven days of treatment, suggesting that PAP is a potent natural agent for wound healing applications.

Catanzano et al. [101] proposed polysaccharide hydrogels combining hyaluronan (HA) in a physically cross-linked alginate (ALG) for dermal wound repair that promoted wound closure after 5 days on a rat model of an excised wound. Abourehab et al. [102] reported the attractive properties of alginate for wound care, such as biocompatibility and high-water absorption, minimizing bacterial contamination and wound secretions. HA also contribute to the management of wounds, regulating tissue hydrodynamics, cell migration and proliferation, and the remodeling and repair process of the extracellular matrix [103]. A sodium alginate-chitosan oligosaccharide-zinc oxide (SA-COS-ZnO) composite hydrogel was fabricated and evaluated for wound healing [104]. The hydrogel showed biocompatibility to blood cells, 3T3 cells, and 293T cells, and antibacterial activity against *E. coli*, *S. aureus*, *C. albicans*, and *Bacillus subtilis*, suggesting a promising approach to manage wound care. Dragostin et al. [105] developed a new chitosan-sulfonamide derivative membrane as a wound dressing biomaterial to test an in vivo study on burn wound models induced in Wistar rats. An improved healing effect with enhanced epithelialization was observed when compared with neat chitosan. Tang et al. [97] developed a tissue-bonded hydrogel based on the incorporation of chitosan, alginate, and polyacrylamide that demonstrated an excellent tissue adhesion, and good mechanical, biocompatibility, and antibacterial properties in the presence of *E. coli* and *S. aureus*, mainly in hydrogels containing chitosan. It suggested that positive-charged amino groups of chitosan damage the bacterial wall by electrostatic interaction with the cytoderm, leading to the release of intracellular fluids [106]. Hydrogels prepared through mixing cellulose and its derivatives with methacrylated gelatin also improved cell adhesion and proliferation for wound healing, favoring wound closure and accelerating wound healing [107]. Considering the studies reporting carbohydrates and their derivatives alone or associated with other biomaterials, free or as a composite, they represent a potent alternative to managing wound care, including burn and infected wounds.

Table 1 summarizes wound healing treatments based on proteins, lipids, and carbohydrates, in different scales (nano, micro, or macro, the type of test (in vitro and/or in vivo), the animal model used in in vivo tests, and the cause of infection presented here as substantial advancements in injury therapies.

## 5. Conclusions

Various proteins are important actors mediating cell–cell and cell–matrix interactions; in view of this, different protein-based formulations, isolated or combined in blends, have been studied as wound healing agents. Lipids, mainly those present in free natural oils and incorporated on nanostructures, films, or dressings, have demonstrated their potential to treat skin’ wounds by different mechanisms. Nevertheless, studies are still needed to clarify molecular mechanisms involved in wound healing and the lipid role in this process, to prove more effective forms to treat wounds. Carbohydrates and their derivatives have also shown potent healing properties to develop new materials for wound care; however, extensive studies are needed to improve their properties for wound dressing applications.

Considering the above reporting proteins, lipids, and carbohydrates for a wound healing treatment, it is possible to state that real-time monitoring of the wound status for feedback is essential for patient recovery. This is especially true for chronic wounds, such as those infected by microorganisms such as *S. aureus*, MRSA, *P. aeruginosa*, and *E. coli*. The relatively new areas of nanotechnology and nanomedicine have been strongly reporting innovative approaches for wound healing and burn infection inhibition. Furthermore, the use of three-dimensional delivery platforms, for example, those based on hydrophilic polymers, for the loading of potent antibacterial substances with intrinsic instability, can be considered the most successfully developed topical formulations for the effective treatment of wound infections.

## Figures and Tables

**Table 1:** Summarizes wound healing treatments based on proteins, lipids, and carbohydrates, in different scales (nano-, micro-, or macro-, the type of test (in vitro and/or in vivo), the animal model used in in vivo tests, and the cause of infection presented here as substantial advancements in injury therapies. Table 1. Recent treatments for wounds based on proteins, lipids, and carbohydrates, in macro-, or micro-, and nanoscale), the type of test (in vitro, in vivo, or both), the animal model (if any), and the cause of infection (if any).

Source	Formulation	Type of Test and Animal Model	Microorganisms Used for Infection	Reference
Proteins				
Silk sericin (SS)	Hydrogel composed by SS, PVA, AZM, and crosslinked with GNP	In vivo study using an infected mouse full-thickness burn model with a 10% total body surface area	*S. aureus*, *P. aeruginosa*, *E. coli*, and *Candida albicans*	[49]
Bovine serum albumin (BSA)	Hydrogel composed by THPS and BSA	In vivo mice model wound infection (female BALB/c mice)	*S. aureus* and MRSA	[50]
Silk	Silk hydrogel, L-carnosine, and curcumin	In vivo wound healing test using streptozotocin tempted diabetic mice	*S. aureus* and *E. coli*	[51]
ε-polylysine	hydrogel fabricated with oxidized alginic acid, dopamine, and antimicrobial peptide ε-polylysine crosslinked with acrylamide	In vivo infected full-thickness wound healing test	Gram-positive and gram-negative bacteria	[52]
Gelatin	Hydrogels based on gelatin methacrylate (GelMA) and dopamine methacrylate (DMA), both of them immersed into zinc nitrate solutions	In vitro tests on NIH-3T3 cells	*E. coli*	[53]
Gelatin	Gelatin hydrogel incorporated with bio-nanosilver (silver nanoparticles from a spent mushroom substrate) functionalized with lactoferrin (LTF)	In vitro anti-biofilm, antibacterial, and cytotoxicity assays	*S. aureus* and *P. aeruginosa*	[54]
Collagen	Bilayer membrane composed of collagen, chitosan, *Aloe vera*, and gelatin	In vivo infected full-thickness wound healing test	Not mentioned	[56]
Fibrin	Fibrin hydrogel incorporating BNN6-loaded mesoporous polydopamine nanoparticles	In vivo wound healing test	MRSA	[57]
Antimicrobial peptide (AMP) from *L. garvieae*	Hybrid hydrogel composed of Pluronic F127 (PF127), ethylenediaminetetraacetic acid (EDTA) loaded liposomes, glutathione (GSH), and the AMP from *L. garvieae.*	In vitro antibacterial and anti-biofilm effects against *S. aureus*; in vivo treatment of MRSA infected mouse wounds	*S. aureus* and MRSA	[59]
Nisin	Nisin incorporated in a composite hydrogel based on natural polysaccharides [gellan gum (GG) and a mixture of GG and alginate)	In vitro antimicrobial test	*S. pyogenes*	[61]
AMP modified hyaluronic acid (HA-AMP)	Hydrogel prepared with the aldehyde group of oxidized-dextran and the amino group HA-AMP through Schiff´s base formation, and platelet-rich plasma	In vitro antimicrobial test and in vivo infected full-thickness wound healing test (diabetic mouse)	*E. coli*, *S. aureus*, and *P. aeruginosa*	[63]
Lipids				
Virgin coconut oil	Oil	In vivo topical application to treat excision wounds in young rats	Not mentioned	[71]
Sunflower/canola oils and canola/linseed oils	Oil blends	In vivo wound healing test in rats	Not mentioned	[74]
*Sapindus mukorossi* seed oil	Hydrogel based on carboxymethyl cellulose (CMC)/hyaluronic acid (HA)/sodium alginate (SA)	In vitro antimicrobial test and in vivo infected full-wound healing test	*Propionibacterium acnes*, *S. aureus*, and *Candida albicans*	[75]
Buriti (*Mauritia flexuosa* L.) oil	Composite chitosan-buriti oil gel	In vitro antimicrobial test and in vivo infected full-wound healing test	*S. aureus* and *Klebsiella pneumoniae*	[76]
Oil extracted from *Opuntia ficus indica L. inermis* (OFI)	Oil	In vitro antimicrobial test and in vivo infected full-wound healing test	*Enterobacter cloacae*, *C. parapsilosis*, *C. shake*, *Penicillium*, *Aspergillus*, and *Fusarium*	[78]
Cocoa butter (solid lipid) and olive oil (liquid lipid)	Nanostructured lipid carriers based on cocoa butter oil, olive oil, and eucalyptus oils	In vitro wound healing properties towards normal human dermal fibroblasts and in vivo wound healing test on a rat burn model	*S. aureus* and *S. pyogenes*	[65]
Oleic acid or linoleic acid	Films based on chitosan containing oleic and linoleic acids, and glycerol	In vivo wound healing test on burned patients	*-*	[84]
Thyme oil	Films based on chitosan and thyme oil	In vitro antimicrobial test	*E. coli*, *K. pneumoniae*, *S. Aureus*, and *P. aeruginosa*	[85]
Lipid components of *Chamaecyparis obtusa* extract	Lipid mixture	In vitro antimicrobial test and in vivo wound healing test	*S. aureus* and *S. pyogenes*	[87]
Peppermint essential oil (PEO)	Nanostructured lipid carriers loaded with PEO	In vitro antibacterial test and in vivo infected wound healing test (mice model)	*S. epidermidis*, *S. aureus*, *Lysteria monocytogenes*, *E. coli*, and *P. aeruginosa*	[88]
Fusidic acid	Nanoengineered lipid-polymer hybrid nanoparticle with chitosan encapsulating the fusidic acid	In vitro antibacterial test	MRSA 33,591 and methicillin-susceptible *S. aureus* (MSSA) 25921	[89]
Fatty acids isolated from the microalgae *Spirulina platensis*	The lipid extracts from *S. platensis* were vectorized using a macroalgal-alginate nanocarrier	In vitro anti-biofilm test	*Candida* species	[90]
Carbohydrates				
Chitosan	Chitosan and Platelet Rich Plasma (PRP)	In vivo infected burn wound test (Wistar rats)	*C. albicans*	[93]
Exopolysaccharide isolated from a marine bacterium *Pantoea* sp. YU16-S3	Hydrogel	In vitro biocompatibility test with dermal fibroblasts and keratinocytes and in vivo wound healing test in rats	Not mentioned	[94]
Exopolysaccharide produced by *Nostoc* sp. Strains PCC7936 and PCC7413	Hydrogel	Wound healing in vitro scratch assay	Not mentioned	[95]
Chitosan	Nanoparticles composed by chitosan, poly(N-vinylpyrrolidone) (PVP), and titanium dioxide (TiO_2_)	In vitro antimicrobial test and in vivo wound healing test (albino rat model)	*E. coli*, *S. aureus*, *P. aeruginosa*, and *B. subtilis*	[98]
Water soluble thymine-modified chitosan (TC) derivatives	TC sponge dressings	In vitro antimicrobial test	gram-negative bacteria, gram-positive bacteria, fungi, drug-resistance bacteria, *P*. *aeruginosa*, and *Acinetobacter baumannii*	[99]
Polysaccharide from *Pimpinella anisum* seeds (PAP)	Gel	In vivo Carrageenan induced paw edema model in mice and topically applied on laser burn lesions	Not mentioned	[100]
Hyaluronan (HA)	Hydrogels combining HA in a physically cross-linked alginate	In vivo Rat model of excised wound	Not mentioned	[101]
Chitosan and alginate	Hydrogel fabricated with sodium alginate-chitosan oligosaccharide-zinc oxide	In vitro biocompatibility and antimicrobial test	*E. coli*, *S. aureus*, *C. albicans*, and *Bacillus subtilis*	[104]
Chitosan-sulfonamide derivative	Membrane	In vivo study on burn wound model induced in Wistar rats	-	[105]
Chitosan and alginate	Hydrogel based on chitosan, alginate, and polyacrylamide	In vitro biocompatibility and antimicrobial test	*E. coli and S. aureus*	[106]
Cellulose and its derivatives	Hydrogel based on the derivatives of cellulose and methacrylated gelatin	In vivo scratch assay wound healing assay and in vivo wound healing test on rats	Not mentioned	[107]

## Data Availability

Not applicable.
